# Indents

**Published:** 1913-12-15

**Authors:** 


### INDENTS.

☞Brief Articles of Technical or Scientific Value will receive notice and comment. Contributions invited.

*Sterilization of Drinking-Water on Small Scale.* Langer states that the taste of the water is not impaired by the method he describes and has found extremely effectual in sterilizing even highly contaminated drinking-water. He uses chlorinated lime (*Chlorkalk*) in the proportion of 0.5 gm. to the liter, mixed with an equal amount of common salt to prevent clumping. After ten minutes the taste of the chemicals is removed by neutralizing with sodium percarbonate. The resulting effervescence lasts for about five minutes during which the lime is transformed into an insoluble compound which can then be filtered out. Thus in less than half an hour the badly contaminated water is made palatable and safe to drink. The slight salty taste left he does not regard as disagreeable. —*Journal A. M. A.*, October 25, 1913.

*Simple Method of Sterilizing Rubber Gloves.* For the past three years I have been using a simplified method of preparing my rubber gloves by which they are rendered sterile and, at the same time, given a coating of talcum, making them easy to adjust. In my hands this method has proved eminently satisfactory, and while some theoretical objections may be raised as regards the completeness of the sterility, results in about three hundred cases have shown absolute safety. My reason for giving this method is that many physicians fail to use rubber gloves in their obstetric work, at least as a routine measure, simply because of the rather laborious methods used in their preparation. My method is as follows:

An 8-ounce bottle is half filled with talc, to which has been added two tablets of mercuric cyanid. The bottle is then filled with 90 per cent. alcohol. This solution is constantly kept on hand in the office. The used gloves are merely washed in hot water, rinsed, placed in a sterile dish and the well-shaken solution poured over them. They are then removed and placed on a sterile towel to dry. The evaporation of the alcohol leaves the gloves completely covered with the dry talc-cyanid powder. They are then wrapped in the towel and placed in the obstetric satchel, while the left-over solution is returned to its original bottle. Gloves prepared in this way have been used by me in cases requiring version, removal of adherent placenta, manual dilatations, etc., and as yet I have never had a case showing septic symptoms of any kind.—Dr. Ray E. Smith, in *A. M. A. Journal.*

*Distilled Water.* Barladean comments on Wechselmann's discovery that certain toxic effects of salvarsan could be avoided by using freshly distilled water. Ordinary distilled water, he found, contained quantities of bacteria and, although killed, by boiling the water, the dead bodies of the bacteria caused disturbance in intravenous injection. Barladean cites the investigations of a number of other workers in this line: Muller examined sixteen specimens of distilled water from the stock of different drug stores and found only two that contained less than 100,000 germs, two containing over 700,000 and one 6,050,000 per c.c. Barladean quotes others to show that not only bacteria but alkali from the glass and metals from the retort, etc., may add to the contamination of distilled water. The influence of even minute amounts of these may be harmful. We know that a thousand millionth part of copper is fatal to a certain fresh water plant, the *Spirogyra*, and another plant, the *Vaucheria*, is even more sensitive to copper ions. He remarks that distilled water is usually kept in large bottles and these bottles can never be thoroughly cleansed. The German Pharmacopeia's requirement for distilled water is that 100 c.c. on evaporation should not leave more than 0.001 gm. residue. This amounts to 0.01 gm. in a liter, and the distilled water is thus merely a dilute solution (1:100,000) of unknown substances. This contamination, Barladean insists, may modify the effect of drugs, even those taken by the mouth. The chemical tests should be supplemented, he declares, by biologic tests with the Spirogyra (to test for metal ions), by bacteriologic tests, as for potable waters, and by testing the electric conductibility of the distilled water.—*Journal A. M. A.*, October, 18, 1913.

*Clean Hands.* The assertion is sometimes made that it is alone the "filthy habits" of the typhoid carrier that make him a public danger. If he could be made to wash his hands, it is alleged, transference of infection would be prevented. Those who regard bacterial cleanliness as simply a matter of careful hand-washing are likely to obtain disappointing results if a recent experiment performed by Cummins[1] is at all indicative of what may occur under ordinary conditions of life. This observer, after dipping the right index-finger in the urine (proved to contain upward of 3,000 million typhoid bacilli per cubic centimeter) of a typhoid carrier, proceeded to carry out measures of cleansing as follows:

1. Rinsed in liquor cresolis compositus solution (approximately 2 per cent.). 2. Then held the finger under the tap, rinsing first in cold, then in very hot water (temperature not recorded). 3. Washed very carefully in about 0.5 c.c. of sterile water in a watch-glass, and plated the whole of the water used for this purpose: Result: Three hundred and thirteen colonies of *Bacillus typhosus* on the plate. 4. After the washing in sterile water mentioned, the tip of the finger was thoroughly soaked in absolute alcohol, allowed to dry, and the washing in sterile water repeated. The "washings" were again "plated". Result: Four colonies of *B. typhosus.*

Even when the fingers are thoroughly rubbed with a towel and the danger of finger infection thereby lessened, it is obvious that the towel in its turn may become infected. The sort of accident that may follow from such conditions is illustrated by another observation of the same author.

On September 26, 1912, 100 c.c. of soup freshly prepared from the "stock pot" was placed in a china bowl, no attempt being made to sterilize the bowl or to cover it from the air. The tip of the experimenter's right index-finger was allowed to come in contact with the urine of Carrier A (proved by plating to contain upward of 3,000 million of the *B. typhosus* per cubic centimeter). The china bowl was then lifted in such a manner that the infected finger came in contact for a moment with the contained soup. The soup was left at room temperature with free access of air and dust to the open bowl. On September 27, enumerated the bacterial contents of the "soup." Result: *B. typhosus* was present apparently in pure culture, numbering 15,500 per cubic centimeter.

Such facts as these add strength to the agitation for better supervision over the conditions of those persons engaged in serving and preparing food for large numbers of people. The action of the Pennsylvania Railroad in providing for the systematic inspection of all of its employees in the restaurant and dining-car systems has already been noted[2]. This example should be followed by the management of other organizations engaged in the handling and serving of food on a large scale. Social clubs and similar bodies, as pointed out by a correspondent recently[3], are often lax in this regard. The supervision of cooks and waiters in dining-cars, hotels, restaurants and clubs is certainly a matter that deserves more attention than it has yet received. "Defective plumbing" is far less important.

---

2. Thrush, M. Clayton: The value of Sanitation as Applied to Railway and Other Large Corporations, The Journal A. M. A., October 4, 1913, p. 1286.

3. Healthy Employees in Kitchen and Dining-Room, Correspondence, The Journal A. M. A., May 10, 1913, p. 1479.

*Another Charge Against Formaldehyd.* Last winter I treated myself for colds with vaccine, hypodermically administered. I had been disinfecting the syringe employed by various methods, usually by placing it in the dry, formaldehyd sterilizer, or by combining this with other familiar methods. I found the pain from the vaccine treatment to be long-continued, in one instance lasting over one month, and noted that a sore spot formed at the place of insertion of the hypodermic needle. Naturally enough, I attributed this mishap to infection, and consequently was more and more careful in cleansing and disinfecting the syringe and the area of skin surrounding the place of insertion—but to no avail. All my efforts to discover the cause of the sores were unsuccessful, until I suspected the formaldehyd chamber, and then the trouble ceased. At present a number of purplish spots still persist at the places where injections were made, which I can attribute to no other cause than formaldehyd. Perhaps the pains in the gums of which patients so often complain after hypodermic injection for the extraction of teeth are in part due to the same cause.

I have become greatly interested in Dr. Grove's "Warning Against the Indiscriminate Use of Formaldehyd Preparations" (published in the *Dental Cosmos* for February, p. 147) in which he relates certain experiments, and concludes that Dr. Buckley's mixture of formaldehyd and tricresol is injurious to the tissues. This statement, if true, is very important, and my own experience, as related above, seems to bear out Dr. Grove's conclusions. On the other hand, teeth treated by the tricresol-formalin method do not have to be extracted in after years as frequently as Dr. Grove's statements would lead one to surmise. For this reason, I am still using Dr. Buckley's treatment, though with some trepidation. Dr. Grove concludes by saying "There are other methods for the correction of these conditions equally satisfactory, and free from the dangerous properties of formaldehyd." It would have been of great interest to know to what other methods Dr. Grove refers. What other method of treatment will abort an abscess as well as that indicated by Dr. Buckley? Is it vaccine therapy, which has been claimed by a manufacturer of vaccines to be most efficient? Is it sodium and potassium? Even if this last combination reached the microbes which have passed beyond the apical foramen, how can it be applied to a distal cavity in a second or third molar, when the tooth is almost too sore to be touched? In many such cases, pain prohibits the application of the rubber dam—indispensable in sodium and potassium treatment—and even the slightest amount of instrumentation is hardly bearable. If Dr. Grove's knows any treatment that will curb the demon of alveolar abscess as readily as Dr. Buckley's mixture, thousands of dental practitioners in my opinion, would be delighted to hear of it from him.—Stewart J. Spence, in *Cosmos.*

*Prevent Silver Oxidation.* Polish the silver and then rub it over with alcohol in which a little collodion has been dissolved. This leaves a thin film of collodion on the surface, which can be easily removed when desired by washing in warm water.—*Scientific American.*

*Ink for Writing on Glass.* Dissolve one ounce of India ink in one and one-half ounces of Sodium Silicate, and apply with pen or suitable brush to clean and dry surface.—*Dental Brief.*

*To Clean Old Dentures, Etc.* Place the articles in a suitable glass or porcelain vessel and cover with water. Add a little soda bicarbonate and then sufficient sulfuric acid to cause a proper effervescence. In a few minutes the plate can be removed cleaner than it is possible to make it by any other practicable process.—M. E. Sanders, in *Dental Brief*, for September.

*Treating Pyorrhea.* Dr. Grieves, in the *Journal of Allied Societies* makes a sensible statement when he says "I seriously object to long drawn out efforts to cure hopelessly suppurating alveolar pyorrhea. Such patients become anemic and sick in spite of the treatment. It were better to extract such teeth and insert suitable prosthetic restorations than to run the risk of serious systemic infection; besides, the process gradually melts away and finally when the teeth are lost the prosthesis is made more difficult.

*Purified Drinking-Water.* The *Bulletin* of the Chicago Department of Health gives the following simple directions for applying the hypochloride method of purification of drinking-water for family or personal use:

Get a few ounces of the best quality of chloride of lime at any drug store and prepare the following:

*Stock Solution.*

| | |
|---|---|
| Chloride of Lime | 1 teaspoonful |
| Water | 1 Quart |

Keep this solution in a tightly stoppered bottle; a Mason jar or a thermos bottle being well adapted for the purposes, the latter especially when one is traveling.

Label the bottle "Stock Solution," show the formula as above, and add the following directions:

"To purify water for drinking purposes, add one teaspoonful of the stock-solution to two gallons of water."

If the water is turbid, strain it through fine muslin before adding any of the stock solution.

After adding stock-solution allow the water prepared for drinking purposes to stand *uncovered* for twenty minutes before using. This allows the gases to escape and makes the water more palatable.—*Brief.*

*Disease Superstitions.* The belief is common among primitive and unlettered people that there is a specific remedy that will cure every disease of the body, if it can only be found.

Ignorant and superstitious people are peculiarly and pathetically susceptible to the persuasions of quacks who profess to have found the healing herb for their particular disease, and will go on squandering money and health after being defrauded a dozen times, because in their simple and pitiful faith they think each time, "Now, maybe, this man has found the real herb that will end my suffering."

This credulity is a matter for patient teaching. The health of the people is a national asset beyond the measure of dollars, but even the economic loss from avoidable sickness and death runs into unbelievable figures. The people must be carefully taught—not casually told—that disease is not an accident, not a dispensation of Providence or the infliction of an evil spirit, but the result of environment and of the mode of living. They must learn that health does not return by magic or by magic compounds; but it must be restored by a personal battle against disease.

Generous physicians, newspapers and journals, and social workers who are giving their time and means to fight the powers to prey and to spread the gospel of health, realize that education is slow. Thousands are saved every year but it will take a long, strong effort to reach all the people with the truth. If ever there was an unselfish effort, and one of supreme importance to the country, it is the battle for national vitality.

What about the national health department at Washington?—*Harper's Weekly and Dental Brief.*

*Undoing the Dentist.* The lower animals and unspoiled man know not the tooth brush, neither do they suffer much from dental caries. The calcium phosphate of the teeth is not dissolved by organic acids. The decomposition products of food are powerless to attack the teeth. The decay of teeth is internal in origin. Calomel internally, syphilis in the blood may decay the teeth, and the microscope demonstrates decay of a tooth to start first in the pulp, beneath the intact enamel.—This statement is not in accord with our present knowledge of the etiology of dental caries.—Editor, *Register*.

Thus says Dr. James R. Mitchell in *The Dietetic and Hygiene Gazette*, March, 1913. And he claims decayed teeth go along with an acid stomach, the excess of HCl abstracting the calcium, and the blood, which *must* have one part of calcium in 10,000, failing to get it from the stomach, takes it from the tissues, the bones and the *teeth*. Too much sugar saps the lime from the blood, and the blood retaliates upon the teeth.

And there you are? The remedy? More lime, less starch and sugar. Demineralized grain products and our sugared but denatured "civilizationizing" of diet are ruining our teeth. Doubtless Dr. Aulde, with his "Chemic Problem in Nutrition," and Mr. McCann, in "Starving America," will agree with Dr. Mitchell.

At all events, if Dr. Mitchell *is* right, and he will make everybody believe he is, it will be the undoing of the dentist. —*Medical Council*, Philadelphia, August, 1913.

*Oral Hygiene Teachers at Loggerheads.*

When doctors disagree the truth of their teaching is seriously discounted by the laity. The discussion upon oral hygiene in the Stomatological Section of the recent Seventeenth International Medical Congress at London disclosed a marked variance in views expressed and a great need of "standardizing" information upon this important subject before presenting it to the public. Dr. L. Cruet recommended a more general diffusion of stomatological ideas among medical men, mothers and all instructors of children. Dr. J. Sims Wallace endorsed this suggestion, and went so far as to advocate a school for teaching medical men dentistry. He considered sugar to be most hurtful to young children, and its excessive consumption to be largely responsible for the prevalence of dental caries. The tooth brush he considered useless. Mr. J. C. Turner, London, did not agree with Dr. Wallace regarding the tooth brush.

As to food, he condemned sticky substances, and advocated the use of coarse meal and dry bread. In his opinion it was useless to insist on filling the teeth of school children, the number was too great and there was not time enough. In a majority of cases they would have to be extracted. Dr. Hutchinson, New York, did not believe in all this faddism about food. The three most important requirements about food were that it should taste nice, give a sense of resistance in mastication, and a comfortable feeling inside when it gets down. He would let a child eat what it wants, when it wants, and as much as it wants.—*London Lancet*, September 13, 1913.—*Brief.*

*Increasing One's Efficiency.* Among the many ways of increasing one's efficiency, especially if the appointment book shows an undue number of vacant hours, is to take up the study of some specialty closely related to one's usual line of practice. Tooth regulating is of the past; except as it relates to simple cases. Orthodontia has taken its place. One who has mastered its science and its details, and has sufficient mechanical skill to arrange or construct the necessary appliances, will find many cases affording opportunity to fill up spare hours profitably to himself and profitably to his patients. It makes for increased usefulness if carried no further than to be able to properly advise patients as to its necessity or importance. Prosthetic dentistry, oral surgery and dental prophylaxis, have in recent years become more important and more progressive. Keeping well in touch with the literature of these specialties makes an operator more efficient, especially if he has learned to closely observe conditions met within daily work, and has learned to bring his own experiences and observations to bear upon the new ideas continually being presented, so as to accurately estimate their value. Efficiency consists not only in doing more work in a given time, and doing it better, with less fatigue to himself and patient, but also in being able to quickly decide what to do and what to leave undone.—Dr. Trueman, in *Brief.*

